# The *in vivo* specificity of synaptic Gβ and Gγ subunits to the α_2a_ adrenergic receptor at CNS synapses

**DOI:** 10.1038/s41598-018-37222-1

**Published:** 2019-02-08

**Authors:** Yun Young Yim, Katherine M. Betke, W. Hayes McDonald, Ralf Gilsbach, Yunjia Chen, Karren Hyde, Qin Wang, Lutz Hein, Heidi Hamm

**Affiliations:** 1 Department of Pharmacology, Vanderbilt University, Nashville, TN 37232-6600 USA; 2 Department of Biochemistry and Mass Spectrometry Research Center, Vanderbilt University, Nashville, TN 37232-6600 USA; 3 Institute of Experimental and Clinical Pharmacology and Toxicology, Faculty of Medicine, University of Freiburg, 79104 Freiburg, Germany; 4 Department of Cell, Development, and Integrative Biology, University of Alabama at Birmingham School of Medicine, Birmingham, AL 35294-3412 USA; 5 Present Address: Geisel School of Medicine at Dartmouth, 1 Rope Ferry Road, Hanover, NH 03755 USA

## Abstract

G proteins are major transducers of signals from G-protein coupled receptors (GPCRs). They are made up of α, β, and γ subunits, with 16 Gα, 5 Gβ and 12 Gγ subunits. Though much is known about the specificity of Gα subunits, the specificity of Gβγs activated by a given GPCR and that activate each effector *in vivo* is not known. Here, we examined the *in vivo* Gβγ specificity of presynaptic α_2a_-adrenergic receptors (α_2a_ARs) in both adrenergic (auto-α_2a_ARs) and non-adrenergic neurons (hetero-α_2a_ARs) for the first time. With a quantitative MRM proteomic analysis of neuronal Gβ and Gγ subunits, and co-immunoprecipitation of tagged α_2a_ARs from mouse models including transgenic FLAG-α_2a_ARs and knock-in HA-α_2a_ARs, we investigated the *in vivo* specificity of Gβ and Gγ subunits to auto-α_2a_ARs and hetero-α_2a_ARs activated with epinephrine to understand the role of Gβγ specificity in diverse physiological functions such as anesthetic sparing, and working memory enhancement. We detected Gβ_2_, Gγ_2_, Gγ_3_, and Gγ_4_ with activated auto α_2a_ARs, whereas we found Gβ_4_ and Gγ_12_ preferentially interacted with activated hetero-α_2a_ARs. Further understanding of *in vivo* Gβγ specificity to various GPCRs offers new insights into the multiplicity of genes for Gβ and Gγ, and the mechanisms underlying GPCR signaling through Gβγ subunits.

## Introduction

G-protein coupled receptors (GPCRs) are the largest and most diverse superfamily of transmembrane receptors that convey signal transduction across cell membranes, and mediate a vast array of cellular responses necessary for human physiology^1–3^. Upon their activation, GTP-Gα and Gβγ subunits are released from the GPCR and interact with various effectors to initiate downstream signaling cascades. Theoretically, 60 different combinations of Gβγ dimers are possible (5 Gβ × 12 Gγ subunits)^4–8^. However, not all theoretical Gβγ dimers exist, are equally expressed, or interact with Gα subunits, receptors, effectors, and downstream signaling factors^5,9–17^. For example, Gβ_1_ and Gβ_4_ dimerize with all Gγ subunits, while Gβ_2_ and Gβ_3_ are unable to dimerize with Gγ_1_ and Gγ_11_^8^. In addition, Gβ_5_ has low-affinity interaction with Gγ subunits^18,19^ and preferentially forms a stable dimer with the RGS R7 subfamily^20–24^. Similarly, Gβ_2_γ_1_ shows a stronger association than Gβ_2_γ_4_^17,25,26^. The expression levels, localizations, and affinities of each Gβ and Gγ subunit influences intracellular signaling cascades through the formation of specific Gβγ dimers and the specificity of each dimer for GPCRs^5,25,27,28^.

Given the diversity seen for the expression and affinity of Gβ and Gγ subunits, as well as the affinity of Gβγ-effector interactions, it is likely that specific dimers could permit specialized roles in signal transduction pathways through association with particular GPCRs. Despite many attempts to understand G protein βγ specificity for particular GPCRs, much remains unclear due to a lack of specific antibodies or other methods of confidently assaying such preferences. Indeed, as yet only *in vitro* data exists which describes Gβγ specificity, and for only a few GPCRs^29–31^. For example, activated α_2a_-adrenergic receptors (α_2a_ARs) are found to interact with Gα_i1_, Gβ_1_, Gβ_2_, Gγ_2_, Gγ_3_, Gγ_4_, and Gγ_7_ as shown by a fluorescence resonance energy transfer (FRET) assay^32,33^ while M_4_ muscarinic receptors interact with Gα_o_, Gβ_3_, and Gγ_4_^34^. Lack of tissue -specific determinants of specificity in heterologous expression systems created a gap between understanding *in vitro* and *in vivo* specificity of G protein βγ. As the interaction Gβγ dimers with particular GPCRs in the CNS may determine their role in regulating synaptic transmission, or their impact in neurological disease and GPCR targeted drug mechanism, further elucidation of G protein specificities *in vivo* is necessary.

α_2a_ARs are G_i/o_-coupled GPCRs^35,36^ that are widely distributed in the peripheral and central nervous systems^37,38^, are expressed in both adrenergic and non-adrenergic neurons, and are located in both pre- and post-synaptic^39^ terminals. Presynaptic α_2a_ARs in adrenergic neurons are called autoreceptors (auto-α_2a_ARs) and act to inhibit exocytosis and prevent norepinephrine release. α_2a_ARs in non-adrenergic neurons are called heteroreceptors (hetero-α_2a_ARs)^37^, and these also inhibit neurotransmitter release. Hetero-α_2a_ARs activity is known to play a role in working memory, hypotension, bradycardia, sedation, analgesia, and hypnosis^37^. Using mRNA *in situ* hybridization and immunohistochemical analysis, auto- and hetero-α_2a_ARs have been found in the locus coeruleus, cerebral cortex, hypothalamus, hippocampus, and amygdala^37,40–43^. Multiple polymorphisms within the *ADRA2A* gene have been identified, which variously increase α_2a_ARs expression and alcohol dependence, reduce glucose-stimulated insulin release and antidepressant responsiveness, and alter memory and behavior^44–46^. In addition, the dysregulation of α_2a_ARs, by increasing the amount of norepinephrine released, enhances fear memory and impairs spatial working memory^47,48^. Though the main mechanism of inhibition of exocytosis is via Gβγ subunits^49–51^, it is unclear which G protein βγs are involved in these downstream signals of α_2a_ARs.

With the development of transgenic mice including Hemagglutinin tagged (HA)-α_2a_ARs knock-in (HA-α_2a_ARs) and FLAG-α_2a_ARs transgenic mice, the physiological implications of α_2a_ARs can be further studied. HA-α_2a_ARs mice were generated utilizing a homologous recombination gene targeting strategy to express HA-α_2a_ARs in the endogenous mouse *ADRA2A* gene locus^52^. Expression and distribution of HA-α_2a_ARs in these mice is identical to those of wildtype mice^52^, as they are expressed in both adrenergic and non-adrenergic neurons which represent both auto- and hetero-α_2a_ARs. Conversely, FLAG-α_2a_ARs transgenic mice express FLAG-α_2a_ARs only in adrenergic neurons, as the transgene is under the control of the dopamine-β-hydroxylase (Dbh) promoter^37^. These mice were then crossed with α_2a_AR knockout (α_2a_ARs KO) mice, such that only FLAG-α_2a_ARs autoreceptors are present. The expression and function of this mice is identical to that of α_2a_ARs autoreceptor^49^. By comparing with the wildtype, FLAG-α_2a_ARs, and α_2a_ARs knock-out mice, the different physiological functions of auto- and hetero-α_2a_ARs were characterized. Auto-α_2a_ARs play a role in bradycardia and hypotension while hetero-α_2a_ARs are involved in anesthetic sparing, hypothermia, analgesia, bradycardia, and hypotension^37^. Given the physiological importance of α_2a_ARs, and the different roles of auto-and hetero-α_2a_ARs, the signaling mechanisms of α_2a_ARs in both adrenergic and non-adrenergic neurons need to be further elucidated.

Together with our previous study quantifying the change in abundance and localization of each neuronal Gβ and Gγ subunit^28^, the differences in physiological functions of auto- and hetero-α_2a_ARs^37^ suggests that the different α_2a_ARs may utilize unique Gβγ dimers to regulate auto- vs. hetero-α_2a_ARs specific downstream signaling pathways. Although Gβ_1_γ_2_ is the most abundant neuronal Gβγ dimer, other Gβγ combinations may be mediating auto- or hetero-α_2a_AR signaling. For example, Gβ_2_γ and Gβ_4_γ dimers may specifically interact with adrenergic and opioid GPCRs^30^. In this paper, we test this hypothesis by using FLAG-α_2a_ARs, HA-α_2a_ARs, α_2a_AR KO, and wildtype mice, together with various biochemical approaches such as a co-immunoprecipitation (co-IP) and a quantitative multiple reaction monitoring (MRM) method to identify and quantify Gβ and Gγ subunits. We measured and compared the interaction of overall (HA-α_2a_ARs) or auto-α_2a_ARs with neuronal Gβ and Gγ subunits for the first time, and depict the *in vivo* Gβγ specificity to auto- and hetero-α_2a_ARs.

## Results

### The interaction of α_2a_ adrenergic receptors and Gβγ

**Figure 1:**
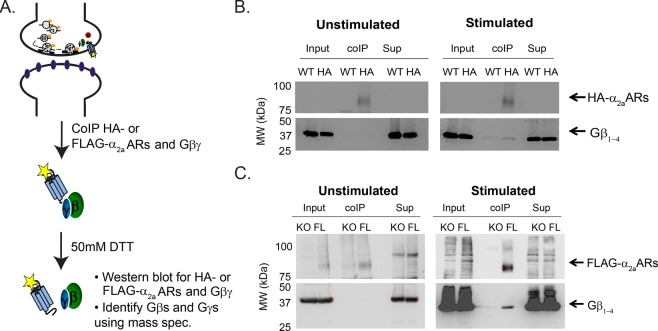
Co-immunoprecipitation of adrenergic α_2a_ receptors and Gβγ. Workflow of co-immunoprecipitation (coIP) experimental protocol (**A**), and representative Western blot of coIP of the HA-α_2a_ARs (**B**) or FLAG-α_2a_ARs (**C**) and Gβs following the resuspension of synaptosomes with unstimulated or stimulated buffers (stimulated, 100 μM epinephrine). Gels are cut out at 50 kDa to separate receptor (HA- or FLAG-α_2a_ARs) and Gβ blots. The exposure times of receptor (HA- or FLAG-α_2a_ARs) blots are 300 secs and 120 secs, respectively. The exposure times of Gβ blots are 300 secs for HA-α_2a_ARs and 100 secs for FLAG-α_2a_ARs coIP. The co-IP lane represents proteins immunoprecipitated with HA or FLAG specific antibodies. HA-α_2a_ARs and FLAG-α_2a_ARs are ~75 kDa while Gβs are ~33 kDa. HA-α_2a_ARs and FLAG-α_2a_ARs interact with Gβγ upon the activation of the receptors (stimulated). Sup: depleted supernatant.

To study the specificity of neuronal Gβγ subunits to synaptic α_2a_ARs, we used brain synaptosomes from wildtype, α_2a_AR KO, HA- and FLAG-α_2a_AR mice. Because no GPCR antibodies are specific enough to co-IP α_2a_ARs and Gβγ, we used HA- and FLAG-α_2a_ARs expressing mice to overcome this limitation. Wildtype and α_2a_-ARs KO mice were used as controls for HA- and FLAG-α_2a_ARs mice. Synaptosomes from these mice were resuspended in a buffer with (stimulated) or without (unstimulated) epinephrine. DSP, a lipid-soluble thiol cleavable crosslinker, was added to ensure the receptor and Gβγ remained intact during co-IP experiments. The synaptosomes were then lysed and co-IPed for HA- or FLAG-α_2a_ARs and Gβγ (Fig. 1A), which was validated by Western blot. Input represents total proteins present in lysate after the preclear while supernatant (Sup) represents what proteins are left in lysate after the co-IP with HA or FLAG specific antibodies (see Materials and Methods for more details). In wildtype and α_2a_ARs KO mice, no α_2a_AR and Gβγ interactions were detected following receptor stimulation (Fig. 1B,C). Here, we detected HA- and FLAG-α_2a_ARs interacting with Gβγ only following α_2a_AR stimulation (Fig. 1B,C).

### Limit of Gβ_1_ detection and quantification

To determine the number of co-IPs needed to detect Gβ and Gγ subunits in our MRM method, we used a serial dilution of purified Gβ_1_γ_1_ and monitored four non-heavy labeled proteolytic peptides of Gβ_1_ to determine the limits of detection and quantitation (LOD/LOQ) (Supplementary Table 1)^53^. Because Gβ_1_γ_1_ is easily purified from the bovine retina, we chose it as our standard. It is used as a control to make sure that our method is running correctly and accurately. Previously, we have validated how each Gβ and Gγ are detected in our quantitative method^28^. Because Gγ_1_ is not present in the brain but only in photoreceptors, we only monitored Gβ_1_ with mass spec. Below 10 pg of Gβ_1_γ_1_, we couldn’t confidently identify the presence of Gβ_1_ in samples. Between 10 pg to 250 pg, we were able to detect Gβ_1_ but total area under the curve (AUC) didn’t increase as the amount of purified Gβ_1_γ_1_ was increased (Supplementary Fig. 1). This suggests that we need more than 250 pg of Gβ_1_ to detect and quantify proteins using our MRM method. We subsequently found using quantitative Western blots, that ~400–700 ng of Gβγ was pulled down with FLAG-α_2a_ARs per half mouse brain used (10 co-IPs/half mouse brain) (data not shown). However, the previous limit of quantification experiment suggests that we need more than 4 ng of Gβγ for quantification^28^. Thus, using a half brain per condition, we can detect and quantify neuronal Gβ and Gγ despite our previously described technical challenges^28^.

### Gβ_2_, Gβ_4_, Gγ_2_, Gγ_3_, Gγ_4_, and Gγ_12_ specifically interact with neuronal α_2a_ adrenergic receptors

**Figure 2:**
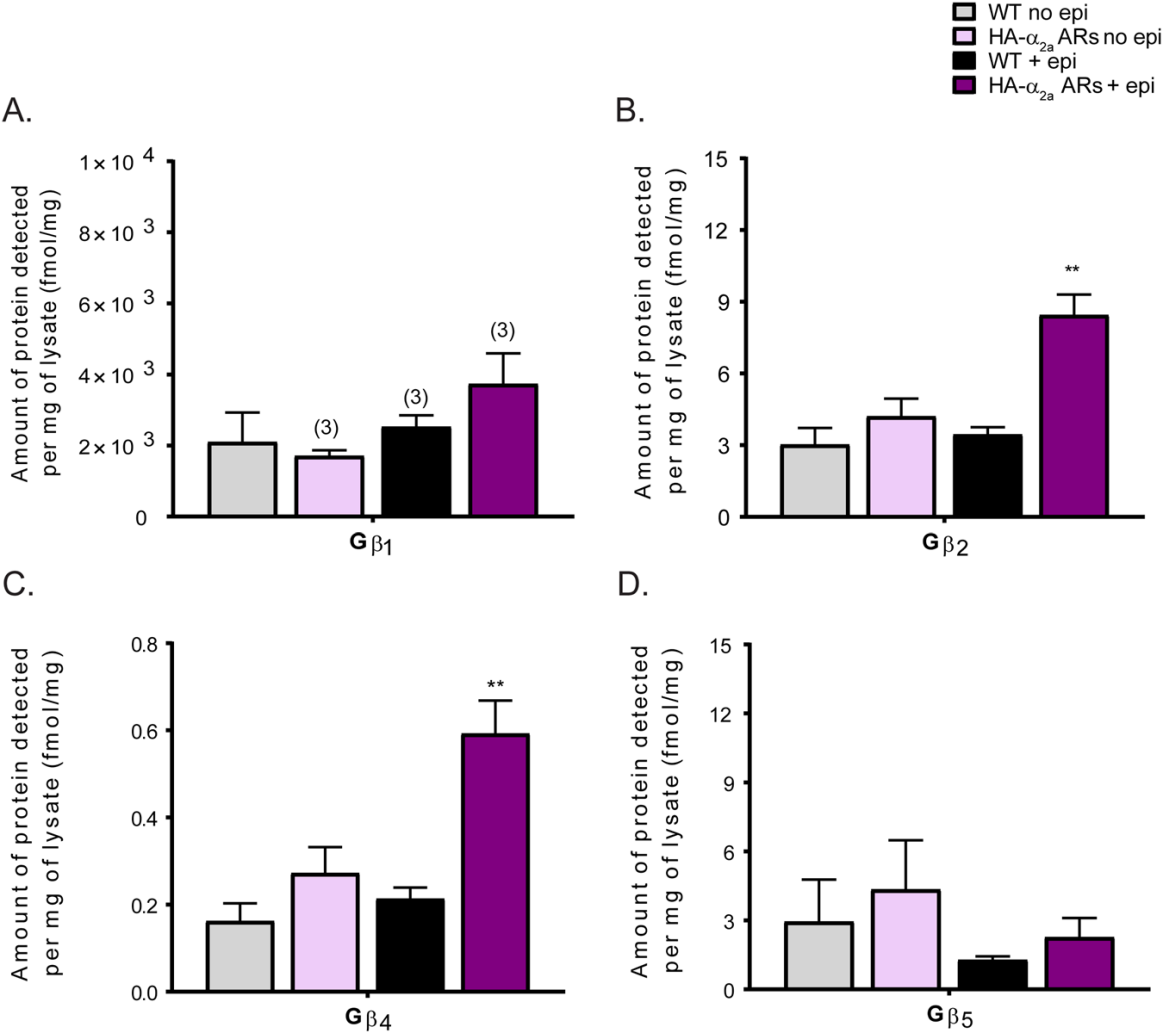
Gβ subunit specificity to α_2a_ adrenergic receptors. Quantification of Gβ subunits interacting with α_2a_ARs in both adrenergic and non-adrenergic neurons (N = 4 unless otherwise noted on the graph with parentheses). Gβ subunits detected (fmol) from quantitative measurements were normalized by the amount of protein (mg), calculated using the volume and the protein concentration of precleared lysate used in co-IPs. We included several controls: unstimulated WT (WT no epi), HA-α_2a_AR (HA-α_2a_AR no epi), and stimulated WT (WT + epi) samples are all controls for the key sample, the Gβ and γ isoforms interacting with HA-α_2a_AR. Gβ_2_ and Gβ_4_ specifically interact with activated α_2a_ARs present in all synaptic terminals. Data were presented as mean ± SEM and compared by a one-way ANOVA, **P < 0.01. *Post hoc* analysis was performed with Tukey’s multiple comparison test.

**Figure 3:**
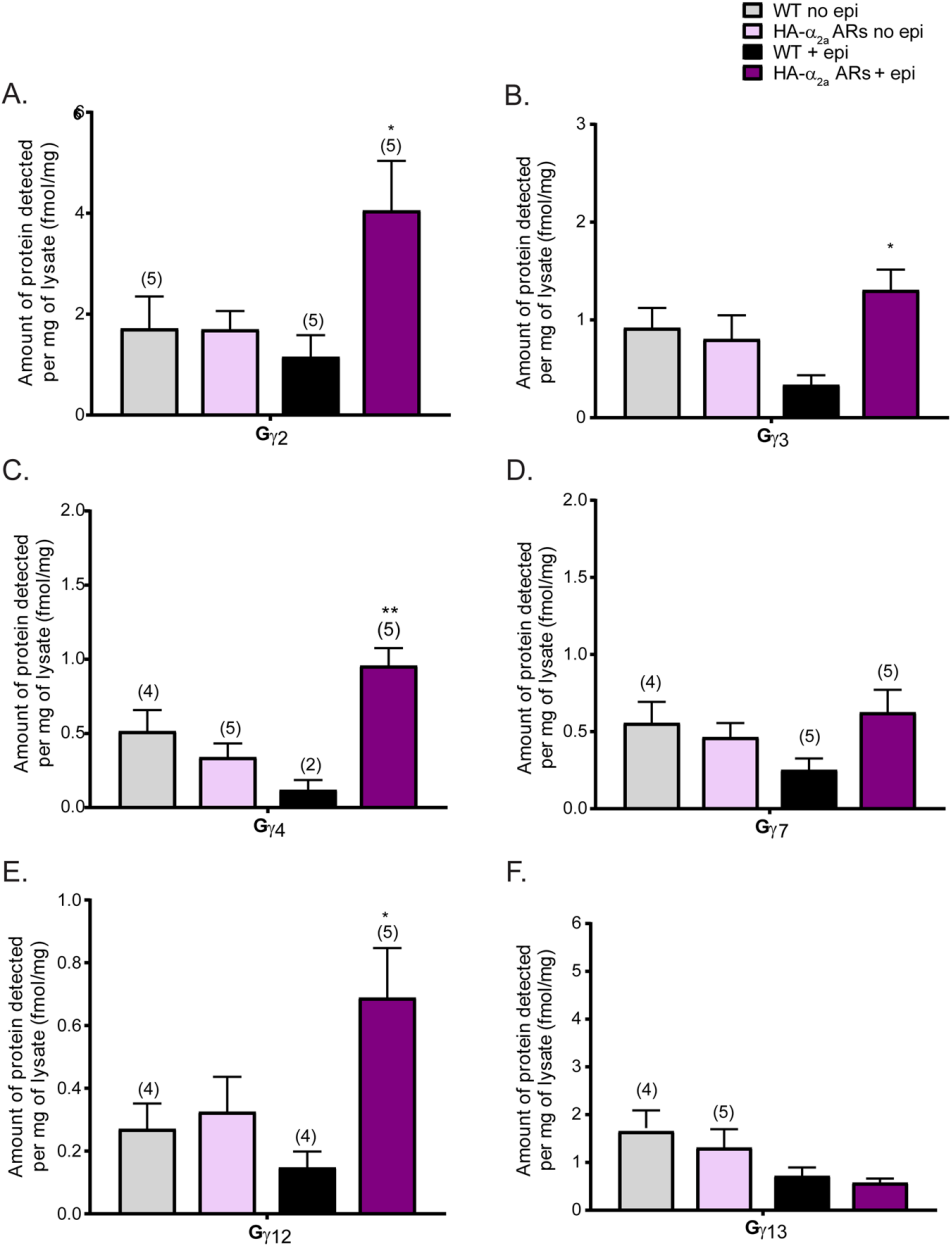
Gγ subunit specificity to α_2a_ adrenergic receptors. Quantification of Gγ subunit interactions with α_2a_ARs in both adrenergic and non-adrenergic neurons (N = 4 unless otherwise noted on the graph). Gγ subunits detected (fmol) from quantitative measurements were normalized by the amount of protein (mg), calculated using the volume of precleared lysate used and the protein concentration of precleared lysate from BCA assay, used in co-IPs. Several controls were run: unstimulated WT (WT no epi), HA-α_2a_AR (HA-α_2a_AR no epi), and stimulated WT (WT + epi) samples. These are all controls for the key sample, the Gβ and γ isoforms interacting with HA-α_2a_AR. Gγ_2_, Gγ_3_, Gγ_4_, and Gγ_12_ specifically interact with HA-α_2a_ARs present in all synaptic terminals. Data were presented as mean ± SEM and compared by one-way ANOVA, *P < 0.05 and **P < 0.01. *Post hoc* analysis was performed with Tukey’s multiple comparison test.

We examined the Gβ and Gγ subunits interacting with α_2a_ARs to distinguish which Gβ and Gγ subunits interact with auto- vs. hetero-α_2a_ARs. In Figs 2 and 3, we applied the quantitative MRM method^28^ to co-IP samples of wildtype (WT) and HA-α_2a_ARs mouse synaptosomes. Using SDS-PAGE gel, we excised Gβ and Gγ bands and added the heavy labeled proteolytic peptides to quantify each neuronal Gβ and Gγ subunit^28^ (see Materials and Methods). Because Gβγ can be sticky, we built in a number of negative controls. To identify nonspecific interactions of Gβ and Gγ subunits, we used both unstimulated WT (WT no epi) and HA-α_2a_AR (HA-α_2a_AR no epi) samples as controls. In addition, we used stimulated WT (WT +epi) samples to detect nonspecific interactions with other receptors (non-HA-α_2a_AR-mediated interactions). Thus the first three conditions in each graph in Figs 2 and 3 were to detect non-specific interactions of Gβγ, while the last detected interaction of Gβγ isoforms with epi-stimulated HA-α_2a_AR.

Gβ_2_ and Gβ_4_ were significantly enriched with HA-α_2a_ARs stimulated with epi (Fig. 2B,C). More Gβ_4_ was detected than Gβ_2_ In contrast, Gβ_5_ did not interact with HA-α_2a_ARs. Next, we examined the specificity of Gγ subunits to α_2a_ARs to determine possible Gβγ dimer interactions with α_2a_ARs. From the 6 detectable and quantifiable neuronal Gγ subunits^28^, Gγ_2_, Gγ_3_, Gγ_4_, and Gγ_12_ were significantly enriched with HA-α_2a_ARs upon epinephrine stimulation (Fig. 3A–C and E). We detected Gγ_2_ > Gγ_3_ ≈ Gγ_4_ > Gγ_12_. Gγ_7_ and Gγ_13_ in stimulated HA-α_2a_ARs + epi samples were equal to, or less, than corresponding control samples, suggesting these Gγs are present nonspecifically (Fig. 3D,F). From the subunits we have detected, we postulate that there may be as many as 8 different combinations of Gβγ dimers *in vivo* (Gβ_2_γ_2_, Gβ_2_γ_3_, Gβ_2_γ_4_, Gβ_2_γ_12_, Gβ_4_γ_2_, Gβ_4_γ_3_, Gβ_4_γ_4_, and Gβ_4_Gγ_12_) which may interact with α_2a_ARs in adrenergic and non-adrenergic neurons. Based on their detection levels, Gβ_2_γ_2_, Gβ_2_γ_3_, and Gβ_2_γ_4_ may be more likely to interact with α_2a_ARs than other Gβγ dimers. Gβ_2_γ_12_, Gβ_4_γ_2_, Gβ_4_γ_3_, Gβ_4_γ_4_, and Gβ_4_Gγ_12_ are less abundant Gβγ dimers interacting with α_2a_ARs. Further biochemical analysis will be needed to validate the presence of these Gβγ dimers and their specificities with α_2a_ARs in both adrenergic and non-adrenergic neurons.

### Gβ_2_, Gγ_2_, Gγ_3_, and Gγ_4_ specifically interact with auto-adrenergic α_2a_ receptors

After identifying the specificities of Gβ and Gγ for α_2a_ARs in both adrenergic and non-adrenergic neurons, we decided to examine the specificity to auto-α_2a_ARs which are only present in adrenergic neurons. In previous studies, auto-α_2a_ARs and hetero-α_2a_ARs were shown to have very different physiological functions^37^. We wondered if these different physiological functions may be mediated by unique Gβ and Gγ specificities for the different receptor types or through specific effector interactions. We again applied a quantitative MRM method to TCA-precipitated and trypsin-digested co-IP samples of α_2a_ARs KO and FLAG-α_2a_ARs mouse synaptosomes.

**Figure 4:**
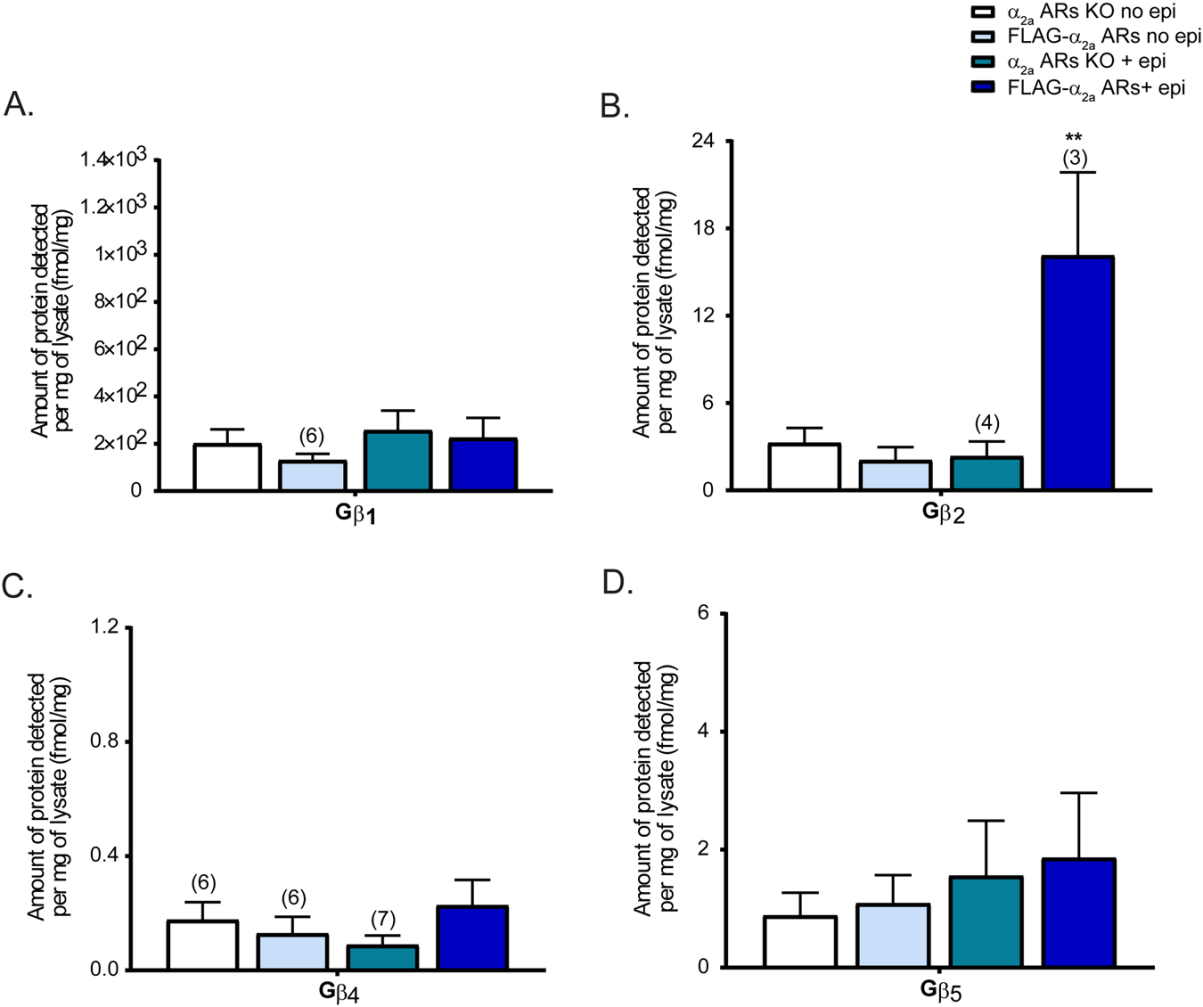
Gβ subunit specificity to auto-α_2a_ adrenergic receptors. Quantification of Gβ subunits interacting with auto-α_2a_ARs (FLAG-α_2a_-ARs**)** in adrenergic neurons (N = 5 unless otherwise noted on the graph). The data were analyzed identical to the study of α_2a_ARs in both adrenergic and non-adrenergic neurons. Unstimulated α_2a_ARs KO (KO no epi), FLAG-α_2a_AR (FLAG-α_2a_AR no epi), and stimulated KO (KO + epi) samples are controls. The difference between these epi-stimulated α_2a_ARs KO and FLAG-α_2a_AR represents the interaction of Gβ isoforms upon auto-α_2a_ARs activation. Gβ_2_ specifically interacts with auto-α_2a_ARs. Data were presented as mean ± SEM and compared by one-way ANOVA, **P < 0.01. *Post hoc* analysis was performed with Tukey’s multiple comparison test.

FLAG-α_2a_ARs only express auto-α_2a_ARs at the sympathetic presynaptic terminal, allowing us to study Gβ and Gγ subunit specificities to autoreceptors uniquely in sympathetic neurons. Similar to the previous experiment, α_2a_ARs KO no epi and FLAG-α_2a_ARs no epi samples were used as controls to identify nonspecific interactions, and α_2a_ARs KO + epi samples were used to detect non-α_2a_ARs associations. Here, Gβ_2_ but not Gβ_4_, showed a significant enrichment with auto-α_2a_ARs (FLAG-α_2a_ARs) (Fig. 4B). Again, Gβ_1_ and Gβ_5_ did not specifically interact with auto-α_2a_ARs upon stimulation (Fig. 4A,D).

**Figure 5:**
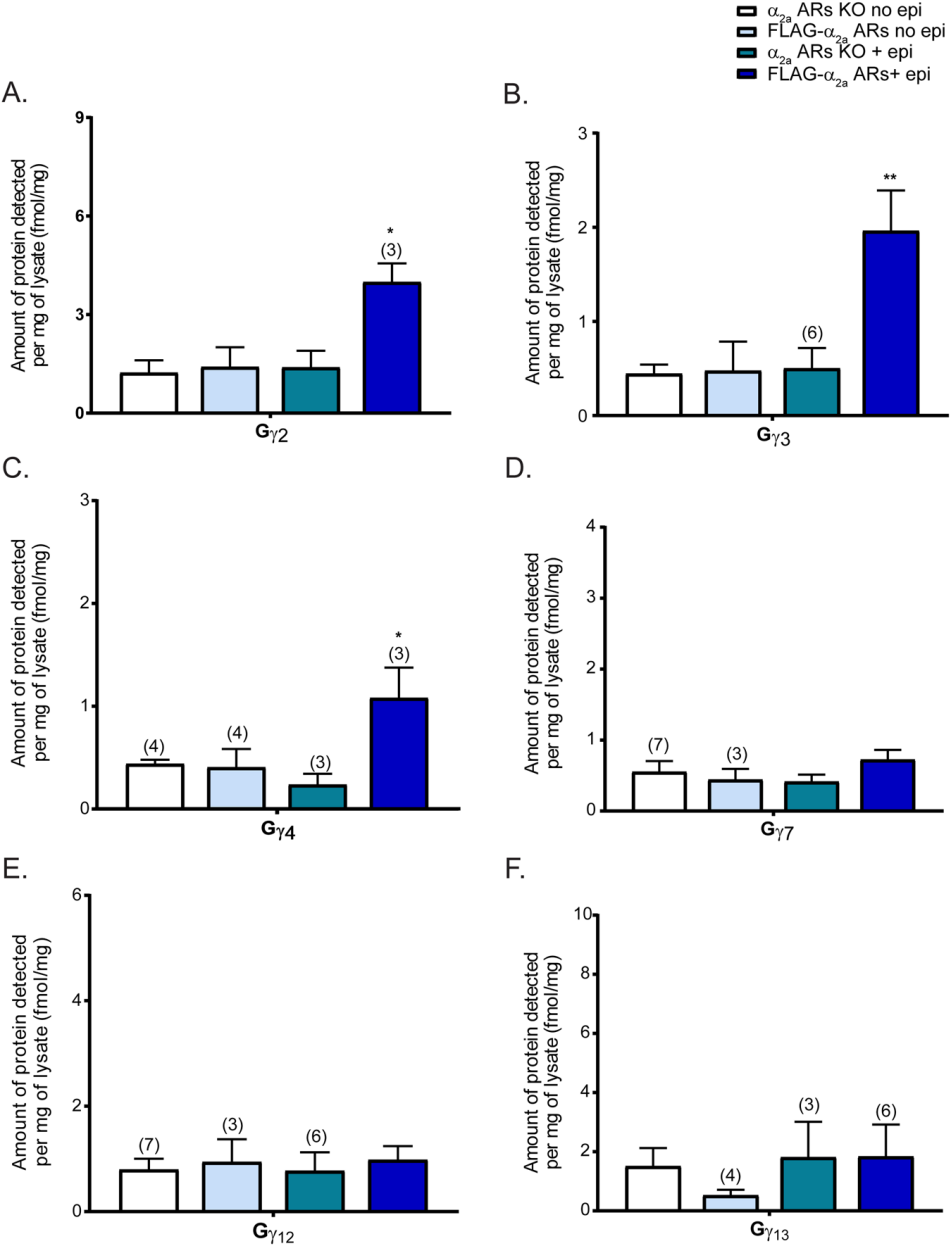
Gγ subunit specificity to auto-α_2a_ adrenergic receptors. Quantification of Gγ subunits interacting with auto-α_2a_ARs on adrenergic neurons (N = 5 unless otherwise noted on the graph). The data were analyzed identical to the study of α_2a_ARs in both adrenergic and non-adrenergic neurons. Unstimulated α_2a_ARs KO (KO no epi), FLAG-α_2a_AR (FLAG-α_2a_AR no epi), and stimulated KO (KO + epi) samples are controls. The difference between these epi-stimulated α_2a_ARs KO and FLAG-α_2a_AR represents the interaction of Gγ isoforms upon auto-α_2a_ARs activation. Gγ_2_, Gγ_3_, and Gγ_4_ specifically interact with auto-α_2a_ARs. Data were presented as mean ± SEM and compared by one-way ANOVA, *P < 0.05 and **P < 0.01. *Post hoc* analysis was performed with Tukey’s multiple comparison test.

In contrast to the 4 Gγ subunits enriched with HA-α_2a_ARs, we were able to detect Gγ_2_, Gγ_3_, and Gγ_4_ enriched with FLAG-α_2a_ARs (Fig. 5A–C). Interestingly, we no longer saw enrichment of Gγ_12_ with FLAG-α_2a_ARs (Fig. 5E) suggesting that Gγ_12_ may be a hetero-α_2a_AR-specific Gγ subunit. As expected from the HA-α_2a_AR study, Gγ_7_ and Gγ_13_ did not interact with FLAG-α_2a_ARs (Fig. 5D,F). Although further validation is necessary, we speculate that Gβ_2_γ_2_, Gβ_2_γ_3_, and Gβ_2_γ_4_ may be the possible Gβγ dimers interacting with auto-α_2a_ARs in sympathetic adrenergic neurons.

### Gβ_4_ and Gγ_12_ may specifically interact with heteroreceptors

Only a subset of Gβ and Gγ subunits from the HA-α_2a_ARs study exhibited specificity to auto-α_2a_ARs, suggesting that hetero-α_2a_ARs may utilize those Gβ and Gγ subunits not associated with auto-α_2a_ARs to regulate unique downstream signaling pathways. Without a transgenic tagged hetero-α_2a_ARs mouse; however, we cannot directly measure the Gβ and Gγ subunits specific to hetero-α_2a_ARs. However, in this study, we can infer the Gβ and Gγ specific to hetero-α_2a_ARs by comparing and subtracting the results of our HA- and FLAG-α_2a_ARs studies. By comparing the Gβ and Gγ subunits detected each set of experiments (which represent overall synaptic α_2a_ARs and presynaptic α_2a_ARs at the sympathetic terminal, respectively), we determined that Gβ_4_ (Figs 2 and 4C) and Gγ_12_ (Figs 3 and 5E) may be heteroreceptor specific. As a result, it is possible that Gβ_2_γ_12_, Gβ_4_γ_2_, Gβ_4_γ_3_, Gβ_4_γ_4_, and Gβ_4_γ_12_ dimers may be left to interact with hetero-α_2a_ARs.

## Discussion

It is well defined that Gβγ dimers are released upon the activation of G_i/o_-coupled GPCRs, such as the α_2a_AR, and act as important signaling units to various downstream signaling cascades to ultimately mediate various physiological functions^54–61^. It is not known whether all 32 possible neuronal Gβγs (combined from the known expression of 4 neuronal Gβs and 8 neuronal Gγs^28^), are functional *in vivo*, however, how such sorting may take place to determine the formation of particular Gβγ dimers is not known, and very little is known of how the specificity of particular Gβγs plays a role in defining the specificity of signaling pathways^5,25,27–34^.

### *In vivo* specificity of α_2a_ARs for Gβγ

In this study, we have addressed the *in vivo* specificity of Gβ and γ interaction with the α_2a_AR using MRM proteomics. We demonstrate that α_2a_ARs preferentially interact with a subset of Gβ and Gγ subunits at synaptic terminals *in vivo*. Neuronal α_2a_ARs (both auto- and hetero-α_2a_ARs) interacted with Gβ_2_, Gβ_4_, Gγ_2_, Gγ_3_, Gγ_4_, and Gγ_12_ while auto-α_2a_ARs interacted with Gβ_2_, Gγ_2_, Gγ_3_, and Gγ_4_ only. These findings suggest that Gβγs may shape signaling pathway specificity and that receptor and Gβγ interactions may be important in determining specific effector interactions.

In our previous study, we found Gβ_1_ as the most abundant Gβ subunit in whole synaptosomes as well as at both pre- and post-synaptic fractions^28^. Interestingly, however, in this study we did not find a statistically significant interaction between Gβ_1_ and HA-α_2a_ARs upon receptor activation (Fig. 2A). Interestingly, we found Gβ_2_ and Gβ_4_ with activated α_2a_AR instead, though there was more than 1,000-fold more Gβ_1_ present at synapses. Despite the low abundance of Gβ_4_ at the membrane^28^, Gβ_4_ binding to α_2a_ARs, as well as the exclusion of the highly abundant Gβ_1_, suggests a high specificity of this interaction. The numbers of receptors and effectors that specifically bind to unique Gβ and Gγ subunits may influence the abundance of certain Gβ and Gγ subunits at the membrane. For example, Gβ_1_ may be specific to other receptors that are more abundant than α_2a_ARs at synaptic terminals. Further studies are needed to determine these specificities, but these findings suggest that each receptor may utilize a unique set of Gβγ dimers to finely regulate receptor-specific downstream signaling.

Moreover, we detected a minor interaction between Gγ_12_ and HA-α_2a_ARs but not with auto-α_2a_ARs (Figs 3 and 5E). Although Gγ_12_ was one of most abundant Gγ subunits at the membrane fraction in our previous study^28^, it was not specifically associated with auto-α_2a_ARs, providing evidence for high specificity of the Gγ_12_ subunit at the hetero-α_2a_ARs. This suggests a Gβ_4_γ_12_ dimer at hetero-α_2a_ARs. In addition, Gβ_5_ showed no specific interaction with α_2a_ARs (Figs 2 and 3D), which supports previous studies that demonstrate it preferentially forms a stable dimer with the RGS R7 subfamily *in vivo* to modulate postsynaptic Gα_i_–mediated signal transduction pathways^20–24^.

As previously addressed^28^, we experienced some technical challenges in detecting and quantifying Gγ subunits with this method. The amount of detected Gγ subunits was not similar to the amount of detected Gβ subunits. This difference may be due to the differences in peptide yield, which could stem from post–translational modifications, sample preparation artifacts, and differences in peptide re-solubilization efficiencies, all of which can lead to systematic errors in quantification^62^. Because of these, we are unable to calculate absolute protein quantities, but we can accurately determine the expression pattern of neuronal Gβ and Gγ subunits and compare within Gβ and Gγ subunits.

### No evidence for pre-coupling of α_2a_AR GPCRs *in vivo*

The association of receptor and G protein prior to receptor activation (“pre-coupling”) has been suggested in some studies, but still remains unclear^1,63–68^. For example, in *in vitro* FRET assay, activated α_2a_ARs were found to interact with Gβ_1_^32,33^. However, in our study using synaptosomes from brain tissue, we do not see significant basal association between α_2a_ARs and Gβ and Gγ. And we see only non-specific interaction between Gβ_1_ and α_2a_AR, even though it is highly abundant pre-synaptically. By contrast, we saw significant interactions of Gβ_2_ and Gβ_4_ with α_2a_ARs, but only after epinephrine activation of α_2a_ARs.

### α_2a_AR autoreceptors vs. heteroreceptors

Our findings suggest that unique Gβγ combination may play specific roles in mediating interactions with receptors. We found different Gβ and Gγ subunits in FLAG-tagged autoreceptors as compared to total HA-tagged α_2a_ARs. This suggests that Gβγ specificities to receptors may change based on the cell type and localization of receptors. We estimate Gβ and Gγ subunit interactions with hetero-α_2a_ARs by subtraction of presynaptic autoreceptor-associated Gβs and Gγs from total HA-α_2a_AR-associated Gβs and Gγs, yielding the finding that Gβ_2_ may be auto-α_2a_AR specific, while Gβ_4_ may be hetero-α_2a_ARs specific. For Gγ subunits, Gγ_2_, Gγ_3_ and Gγ_4_ were determined to be auto-α_2a_ARs specific, while Gγ_12_ was hetero-α_2a_ARs specific. (Table 1). Overall, hetero-α_2a_ARs may associate with G protein heterotrimers paired with Gβ_4_γ_12_ to mediate hetero-α_2a_AR-specific phenotypes such as sedation and anesthetic sparing^37^. One difference between these two mice is that heteroreceptors may be found either pre- or post-synaptically, whereas autoreceptors are only pre-synaptic.

**Table 1:** Gβ and Gγ specificities to hetero-α_2a_ARs.

G proteins	α_2a_ ARs	Auto-α_2a_ ARs	Hetero-α_2a_ ARs (estimated)
Gβ_2_	++	++	−
Gβ_4_	+	−	+
Gγ_2_	+++	+++	−
Gγ_3_	++	++	−
Gγ_4_	+	+	−
Gγ_12_	+	−	+

The number of + denotes abundance. +: interaction with receptor detected; −: no interaction was detected.

We were not able to separate these two populations of heteroreceptors to determine whether this localization makes a difference. We were able to compare the results of these two studies side-by-side as similar levels of proteins were detected for most Gβ and Gγ subunits, however, one limitation of our studies is that we were unable to determine the differences in co-IP efficiency of HA- and FLAG- antibodies and the number of receptors in digested samples to calculate the relative Gβ and Gγ enrichment with hetero-α_2a_ARs. Again, future studies with refined methodologies are needed to determine the functional consequences of identified specificities.

Because HA-α_2a_ARs represent both auto- and heteroreceptors and are found throughout the brain, we did not specify the neuronal type nor the location of receptors in the synaptosomes. Gβ_2_ and Gβ_4_ were previously identified to interact with α_2a_ARs^30^, and in this study these Gβ subunits are identified to interact with Gγ_2_, Gγ_3_, Gγ_4_, Gγ_12_ subunits. The rank order of Gγ specificity to overall neuronal α_2a_ARs is similar to the Gγs found in whole and fractionated synaptosomes in the previous study^28^. It still remains unclear which Gγ subunits associate with each Gβ subunit. Though the rules for specificity determination are unknown, we assume that multiple factors affect the specificity: the preference of these Gβ subunits for Gγ subunits, the localization of receptors, and effector availability. The protein abundance and location of Gγ subunits will affect the Gβγ dimerization and their specificity to α_2a_ARs.

### Gβ and Gγ subunit specificity to α_2a_ARs studied *in vitro*

Numerous *in vitro* studies have attempted to determine the specificity of Gβγ dimerization and their selectivity in interacting with various GPCRs and effectors^11,69,70^. Similar to our observations, Gβ_2_, Gβ_4_, Gγ_2_, Gγ_3_, and Gγ_4_ were previously shown to be strongly associated with α_2a_ARs^32,71^. Using FRET, Gibson and Gilman demonstrated that endogenous α_2a_ARs preferentially stimulated Gα_i1_ heterotrimers paired with Gβ_1_ or Gβ_4_, and Gα_i3_ heterotrimers paired with Gβ_2_^32^. They also found that Gβ_2_ association permitted 2-fold higher receptor activation, which was lost when Gβ_2_ was replaced with Gβ_1_. This result and our studies suggest that α_2a_ARs with Gα_i3_β_2_γ heterotrimers may be most likely to be present at the *in vivo* synaptic terminals. Moreover, Gβ_2_γ and Gβ_4_γ dimers were determined to interact with adrenergic and opioid GPCRs, while Gβ_1_γ and Gβ_3_γ dimers, particularly Gβ_1_γ_3_ and Gβ_3_γ_4_, may preferentially couple with somatostatin and muscarinic M4 GPCRs^29–31^. However, no specificity was identified based on the localization of receptors. In addition to the identify of Gα and Gγ subunits, the localization of receptor may play a role in α_2a_AR selectivity of Gβ_2_ and Gβ_4_ over Gβ_1_. Depending on the localization of receptor, α_2a_ARs may also preferentially interact with specific effectors. Based on our results and previous biochemical studies, Gβ_2_γ_2_, Gβ_2_γ_3_, and Gβ_2_γ_4_ may be auto-α_2a_ARs specific, while Gβ_4_γ_12_ may be hetero-α_2a_ARs specific.

Other *in vitro* G protein specificity studies^71–74^ depict a different Gβ and Gγ specificity than seen in our study. The gap between *in vitro* and *in vivo* detection of G protein specificity may be explained by tissue-specific determinants of specificity that are not present in heterologous expression systems, or difference in expression and availability of Gβ and Gγ subunits for *in vitro* studies. It is clear that Gβγ subunits are sticky, and this is why we provided multiple controls for non-specific effects. Future studies will be needed to address these differences.

### Role of Gα subunits in determining Gβγ specificity to α_2a_AR receptors

In addition to Gβγ, Gα may also define the selectivity of G_i/o_–coupled GPCRs such as α_2a_ARs. Unlike Gα_s_, much less is known about how GPCRs selectively activate inhibitor Gα_i1–3_ and Gα_o_ subunits. Recent cryo-electron microscopy (cryoEM) studies reporting the structures of G_i/o_ bound GPCRs, such as μ-opioid^75^, adenosine A_1_^76^, 5HT_1B_^77^, and light receptor rhodopsin^78^, determine the interaction of these receptors with G_i_ or G_o_ and suggest the conformational re-arrangements on the GPCR cytoplasmic site may affect the binding of specific G proteins. Interestingly, they found different interactions of G_i/o_ bound GPCRs and Gβ subunits^79^. However, the role of Gβγ in GPCRs-G protein specificity is unclear in these studies due to the modification of the proteins and the resolution of cryoEM structures. Moreover, the studies of GABA_B_ heteromeric receptors with GABA_B1_ and GABA_B2_ have suggested hetero-dimerization of GPCRs may also affect the binding interactions of Gβγ with the receptor^80,81^. Further studies are needed to determine how Gα subunits affect the specificity of Gβγ.

As a G_i/o_–coupled GPCR, α_2a_ARs couple to Gα_i1–3_ and Gα_o1–2_. In a previous study by Richardson and Robishaw, Gα_i_-containing heterotrimers were highly coupled to α_2a_ARs^71^. Further, Gα_i_ subunits were demonstrated to mediate sedative anesthetic-sparing effects, but not inhibition of evoked release^82^, and Gαi_1_ were found to preferentially associate with Gβ_1_γ_3_ over Gβ_1_γ_1_ or Gβ_1_γ_10_^71^. This suggests that Gα−mediated selectivity additionally contributes to the specificity of α_2a_AR signaling through G proteins and their physiological functions. Further studies will be needed to understand the specific associations of Gα subunits with the Gβ and Gγ subunits observed here and their roles in known α_2a_AR-mediated physiological effects.

## Conclusions

With the quantitative MRM method^28^, we now can further elucidate the *in vivo* Gβ and Gγ specificities to other GPCRs as well as Gβγ effectors, and validate previous *in vitro* studies of the Gβγ dimerization and their selectivity in interacting with various GPCRs and effectors^11,69,70^. In the CNS, numerous Gβ and Gγ subunits exhibit interesting subcellular localizations^28,83^. We do not yet fully understand the importance of these localizations and their physiological role, however. This study begins to piece together the puzzle why multiple different isoforms of Gβ and Gγ subunits exist. Further efforts and development of tools, such as knockout or tissue-specific knockout animals, will be needed to determine the specificity and roles of each unique Gβγ dimer in regulating various GPCR signaling cascades, and their impacts on neurological diseases and GPCR targeted drug mechanisms. Eventually this will allow us to determine how cells precisely regulate multiple downstream mechanisms to modulate signal intensity and specificity.

GPCR specificity to G proteins is defined by the Gα subunit preferred by a given GPCR. Whether GPCRs also have preference for Gβ and Gγ subunits is not well investigated. Here, we measured the *in vivo* specificity of presynaptic α_2a_ARs to a subset of neuronal Gβ and Gγ subunits using a previously published proteomic approach. We found that Gβγ dimers, other than the most abundant Gβ_1_γ_2_, are also involved in α_2a_ARs-mediated signaling cascades *in vivo*. In addition, auto- and hetero-α_2a_ARs exhibit specificity to different Gβ and Gγ subunits. The variety of potential Gβγ dimers identified implies that the specificity of Gβγs to signaling pathways could be in part mediated through the receptors and their locations on particular types of neurons.

## Materials and Methods

See supplementary for more details.

### Animals

Adult, male HA- and FLAG-alpha2a adrenergic receptors (α_2a_ARs), α_2a_ARs knockout (KO), and wildtype mice^37,52^ were used. All animal handling and procedures were conducted in accordance with the Care and Use of Laboratory Animals of the National Institutes of Health and approved by the Vanderbilt Institutional Animal Care and Use Committee.

### Drugs

Epinephrine (catalog E4642), prazosin (catalog P7791), and propranolol (catalog P0884) were purchased from Sigma-Aldrich.

### Antibodies

Mouse anti-HA-agarose (Sigma, A2095), mouse anti-FLAG (Sigma, F3165) mouse anti-HA (Covance, 901514, 1:750), rabbit anti-FLAG (Sigma, F7425, 1:100), and rabbit anti-Gβ (Santa Cruz, sc-378, 1:10,000 and 1:5000) were used.

### Synaptosome

Crude synaptosomes were isolated from mouse brain tissue, as described previously^53,84,85^ and stimulated with 100 µM epinephrine (epi). This mimics the local synaptic concentration of epinephrine and it is a commonly used concentration in alpha2a adrenergic receptor studies^86–88^. They were frozen in liquid nitrogen and stored at −80 °C.

### Co-immunoprecipitation (Co-IP)

Crude synaptosomes were gently resuspended in 4 mL of RIPA buffer using a 25-gauge needle to lyse membranes and diluted to 1 mg/ml. Homogenates were centrifuged to separate the triton-soluble and insoluble fractions. Triton-soluble fractions were used for co-IP by incubating with either an anti-HA or FLAG antibody and Protein G agarose beads overnight. For elution, 100 µL of 1X sample buffer with DTT and 5% βME were used for HA-α_2a_ARs and wildtype samples while 15.09 µg FLAG peptide was used for FLAG-α_2a_ARs and α_2a_ARs KO samples. Elutants were TCA precipitated and resuspended in 100 µL of 1x sample buffer with DTT and 5% βME. All samples were stored at −80 °C freezer for Western blot or MRM analysis.

### Immunoblot analysis

To examine the results of IP, Western blot analysis was performed on equal volumes of input, co-IP, and supernatant samples using 10% SDS-PAGE gels. Using Western Lightning™ Chemiluminescence Reagent Plus (Perkin-Elmer) and Bio-rad Western blot imager, Western blots were developed.

### Heavy labeled peptide cocktail

A heavy labeled peptide cocktail was made as described previously^28^.

### Quantitative MRM of Gβ and Gγ subunits

Co-IP samples containing Gβ and Gγ subunits were separated, digested, and analyzed by a TSQ Vantage triple quadrupole mass spectrometer (Thermo Scientific)^28^. To allow comparisons between G proteins co-IPed from multiple mice, quantitative Gβ and Gγ subunits detected (fmol) were normalized by the amount of protein (mg) used in co-IPs. The amount of protein used in co-IPs was calculated using the volume of precleared lysate used and the protein concentration of precleared lysate from BCA assay.

### Statistical analysis

One-way analysis of variance (ANOVA) with a Tukey post hoc test was used to account for differences in protein expression of Gβ and Gγ subunits (^*^p < 0.05, ^**^p < 0.01, ^***^p < 0.001). All statistical tests were performed using GraphPad Prism v.7.0 for Windows, (GraphPad Software, La Jolla, California, USA, www.graphpad.com).

## Supplementary information

The in vivo specificity of synaptic Gβ and Gγ subunits to the α2a adrenergic receptor at CNS synapses

## Data Availability

All data generated or analyzed during this study are included in this published article (and its Supplementary Information files).
