# Author Correction: The *in vivo* specificity of synaptic Gβ and Gγ subunits to the α_2a_ adrenergic receptor at CNS synapses

**DOI:** 10.1038/s41598-020-59022-2

**Published:** 2020-02-14

**Authors:** Yun Young Yim, Katherine M. Betke, W. Hayes McDonald, Ralf Gilsbach, Yunjia Chen, Karren Hyde, Qin Wang, Lutz Hein, Heidi E. Hamm

**Affiliations:** 1 Department of Pharmacology, Vanderbilt University, Nashville, TN 37232-6600 USA; 2 Department of Biochemistry and Mass Spectrometry Research Center, Vanderbilt University, Nashville, TN 37232-6600 USA; 3 Institute of Experimental and Clinical Pharmacology and Toxicology, Faculty of Medicine, University of Freiburg, 79104 Freiburg, Germany; 4 Department of Cell, Development, and Integrative Biology, University of Alabama at Birmingham School of Medicine, Birmingham, AL 35294-3412 USA; 5 Present Address: Geisel School of Medicine at Dartmouth, 1 Rope Ferry Road, Hanover, NH 03755 USA

Correction to: *Scientific Reports* 10.1038/s41598-018-37222-1, published online 08 February 2019

The original version of this Article contained an error in the author name Heidi E. Hamm, which was incorrectly given as Heidi Hamm.

This has now been corrected in the PDF and HTML versions of the Article, and in the accompanying Supplementary Information file.

