# Supplementary material for: The in vivo specificity of synaptic Gβ and Gγ subunits to the α2a adrenergic receptor at CNS synapses
Source: Sci Rep. 2019 Feb 8;9:1718. doi: 10.1038/s41598-018-37222-1 (PMC6368627; doi:10.1038/s41598-018-37222-1)
Supplement: Supplementary file 1 — The in vivo specificity of synaptic Gβ and Gγ subunits to the α2a adrenergic receptor at CNS synapses [file 41598_2018_37222_MOESM1_ESM.pdf]

Supplementary Information for

The *in vivo* specificity of synaptic Gβ and Gγ subunits to the α_{2a} adrenergic receptor at CNS synapses

Yun Young Yim[1], Katherine Betke[1,5], W. Hayes McDonald[2], Ralf Gilsbach[3], Yunjia Chen[4], Karren Hyde[1], Qin Wang[4], Lutz Hein[3], and Heidi Hamm[1*]

Department of Pharmacology, Vanderbilt University, Nashville TN 37232-6600
[2]Department of Biochemistry and Mass Spectrometry Research Center, Vanderbilt University, Nashville TN 37232-6600
[3]Institute of Experimental and Clinical Pharmacology and Toxicology, Faculty of Medicine, University of Freiburg, 79104, Freiburg, Germany
[4]Department of Cell, Development, and Integrative Biology, University of Alabama at Birmingham School of Medicine, Birmingham, AL 35294-3412
[5]Currently at Geisel School of Medicine at Dartmouth, 1 Rope Ferry Road, Hanover NH, 03755

Dr. Heidi Hamm
Email: heidi.hamm@Vanderbilt.Edu

Material and Methods

Animals. Adult, male HA- and FLAG-alpha2a adrenergic receptors (α_{2a}ARs), α_{2a}ARs knockout (KO), and wildtype mice[1,2] were decapitated, and brain tissues were immediately homogenized to produce crude synaptosomes as described below. To minimize post-mortem differences, all tissues were processed in parallel. All animal handling and procedures were conducted in accordance with the Care and Use of Laboratory Animals of the National Institutes o Health and approved by the Vanderbilt Institutional Animal Care and Use Committee.

Drugs. Epinephrine (catalog E4642), prazosin (catalog P7791), and propranolol (catalog P0884) were purchased from Sigma-Aldrich.

Antibodies. For the immunoprecipitation, Mouse anti-HA-agarose (Sigma, A2095) and mouse anti-FLAG (Sigma, F3165) were used. For the Western blot analysis, mouse anti-HA (Covance, 901514, 1:750), rabbit anti-FLAG (Sigma, F7425, 1:100), and rabbit anti-Gβ (Santa Cruz, sc-378, 1:10,000 and 1:5000) were used. HRP-conjugated secondary antibodies were obtained from Perkin-Elmer, Abcam, and Jackson Immunoresearch and used at the following dilutions: goat anti-rabbit (1:10,000), goat anti-mouse (1:10,000), Abcam anti-mouse light chain specific (1:5000), and mouse anti-rabbit light chain specific (1:7,500).

Synaptosome preparation. Crude synaptosomes were isolated from mouse brain tissue, as described previously [3-5]. Briefly, whole brains were sectioned at the midsagittal plane to equally divide the right and left hemispheres. Both sections were separately homogenized in 20 mL of a 0.32 M sucrose solution (0.32M sucrose, 4.2 mM HEPES pH 7.4, 0.1 mM $CaCl_2$, 1 mM $MgCl_2$, 1.54 μM aprotinin, 10.7 μM leupeptin, 0.95 μM pepstatin, and 200 μM PMSF). Homogenates were centrifuged at 1000 x g and 4°C for

10 min, and supernatants containing synaptosomes (S1) were transferred to clean conical tubes. Pellets were resuspended in 20 mL of 0.32 M sucrose solution and centrifuged again. Pellets were discarded. Supernatants (S1) were combined and centrifuged at 10,000 x g and 4°C for 20 min to produce the crude synaptosome pellet (P2). One whole brain yielded two crude synaptosome pellets (P2).

Stimulation of Synaptosome. Crude synaptosomes (P2) were gently re-suspended in 2mL of resuspension buffer (10X DPBS (26.7mM KCl, 14.7mM KH_2PO_4, 1379mM NaCl, 80mM Na_2HPO_4•$7H_20$, pH 6.8-7), 1μM prazosin, and 1μM propranolol, transferred to 6ml culture vials, and placed on ice. As epinephrine (epi) is not an α_{2a}ARs selective agonist, we used prazosin and propranolol to block off-target effects from non-α_{2a}ARs adrenergic receptors. For each set of crude synaptosomes per whole brain, one was used as an unstimulated control while the other was stimulated to examine α_{2a}ARs and G proteins (Gβ and Gγ) selectivity. For the control condition, 4mL of resuspension buffer was added to each vial and gently mixed. To stimulate α_{2a}ARs, 2ml of resuspension buffer was added to each vial first. Then, 2mL of stimulation buffer (resuspension buffer containing 200μM epi) were added to each vial to make the final concentration of epi to be 100μM in the stimulation condition and gently mixed. All samples were placed in a 37°C water bath for 2 minutes (mins) and incubated with 2mM of the lipid soluble, thiol cleavable crosslinker, 3,3'-dithiobis [sulfosuccinimydlypropinate] [6] (Pierce, 22585) for 2 hours (hrs) on ice. After 2 hrs, crosslinking reactions were quenched with 20mM Tris, pH 7.4 for 15 mins on ice. All samples were centrifuged at 10,000 x g at 4°C for 20 mins to regenerate the P2 pellet,

then washed with 4mL of resuspension buffer, and centrifuged again. Stimulated and non-stimulated crude synaptosomes were frozen in lipid nitrogen and stored at -80°C.

Synaptosome lysate. Crude synaptosomes were gently resuspended in 4 mL of RIPA buffer (50 mM Tris, pH 7.4, 150 mM NaCl, 0.1% SDS, 1% sodium deoxycholate, 1% Triton X-100, 1 mM EDTA, 1.54 μM aprotinin, 10.7 μM leupeptin, 0.948 μM pepstatin, and 200 μM PMSF) using a 25 gauge needle to lyse membranes. Lysate protein concentrations were determined with a BCA assay (Pierce) and diluted to 1 mg/mL using RIPA buffer. The diluted homogenate was placed on a rotator for 1 hr and maintained at 4°C. Homogenates were transferred to 2 mL Eppendorf tubes and centrifuged at 14,000 rpm at 4°C for 10 min to separate the triton-soluble and insoluble fractions. Supernatants, the triton-soluble fractions, were collected. Supernatant protein concentrations were determined with a BCA assay.

Co-immunoprecipitation. Triton-soluble fractions were transferred to clean 2mL Eppendorf tubes and precleared for 1 hr at 4°C with 50μL of Protein G agarose beads (Pierce 20398). After the preclear, samples were centrifuged at 5,000 x g for 2 mins to pellet the beads. Protein concentrations of precleared lysates were determined with a BCA assay. Then, 300μL of precleared lysate per condition per genotype were saved as "inputs," and mixed with 100μL of 4X sample buffer containing dithiothreitol (DTT) and 5% βME. Inputs were heated at 70°C for 5 mins and frozen at -80°C for Western blot analysis. The remainders of the precleared lysates were aliquoted to 1.5ml Eppendorf tubes. Precleared lysates from HA-α_{2a}ARs and wildtype mice were aliquoted using 1ml per tube, while lysates from FLAG-α_{2a}-ARs and α_{2a}-ARs KO mice were aliquoted using 400ul per tube. Lysates were incubated with either an anti-HA or FLAG co-

immunoprecipitation (coIP) antibody for 1 hr at 4°C. After 1 hr, 30μL of Protein G agarose beads was added to anti-FLAG coIP tubes only. All samples were rotated at 4°C overnight. The following day, all tubes were centrifuged at 5,000 x g for 2 mins to pellet the beads, and 300μL of supernatant per condition per genotype were transferred to clean, "supernatant" labeled 1.5mL Eppendorf tubes. Supernatants were mixed with 100μL of 4x sample buffer containing DTT and 5% 2-mercaptoethanol (βME), heated at 70°C for 5 mins, and frozen at -80°C for the Western blot analysis. Remaining supernatant was combined per condition and genotype and stored at -80°C. Beads were washed twice for 5 mins at 4°C with 500μL of coIP buffer (50mM Tris, pH 7.4, 150mM NaCl, 0.5% Triton X-100, 1.54μM aprotinin, 10.7μM leupeptin, 0.948μM pepstatin, and 200μM PMSF) and pelleted at 1,000 x g for 2 mins. Following the second wash, beads were resuspended in 500μL of coIP buffer, transferred to clean 1.5mL Eppendorf tubes, rotated for 5 mins in 4°C, and pelleted at 5,000 x g for 2 mins. Supernatants were aspirated, and beads were ready for the elution. (Fig. 1A).

Elution and trichloroacetic acid (TCA) precipitation of co-IPs. Two different elution methods were used, depending on genotype. For HA-α_{2a}ARs and wildtype samples, 100μL of 1X sample buffer with DTT and 5% βME were added to the samples, vortexed, and heated at 70°C for 5 mins. For FLAG-α_{2a}ARs and α_{2a}ARs KO samples, stock aliquots of 5mg/ml FLAG peptide (Sigma, F3290) were diluted with TBS to 0.377mg/ml, and 40μL the peptide was added to the co-IP beads for a final amount of 15.09μg FLAG peptide. Samples were placed on a vortex shaker at medium speed for 30 mins and centrifuged at 5,000 x g for 2 mins to pellet beads. Eluents were transferred to clean 1.5mL Eppendorf tubes. The elution was repeated a second time, and the eluents were

pooled together with the first elution. For Western blot analysis, one tube per condition per genotype was saved separately. Remaining eluent from same conditions was pooled per genotype and TCA precipitated to concentrate the G proteins for MRM analysis. One tube was saved per condition per genotype as samples were incubated with 25% TCA (Sigma, T6399) on ice for 30 mins before being centrifuged at 14,000 rpm at 4°C for 30 mins. Following the centrifugation, supernatants were removed and pellets were washed with 500μL of cold acetone and centrifuged again at 14,000 rpm, 4°C for 15 mins before carefully aspirating off supernatants. The wash step was repeated twice and at the last centrifugation, supernatants were carefully removed using a pipette and dried down by the speed-vac for 5 mins. Dried pellets were resuspended in 100μL of 1x sample buffer with DTT and 5% βME, and heated at 70 °C for 5 mins. All samples were stored at -80°C freezer for Western blot or MRM analysis.

Immunoblot Analysis. To examine the results of IP, Western blot analysis was performed on equal volumes of input, co-IP, and supernatant samples using 10% SDS-PAGE gels. Input and supernatant samples were diluted 1:1 with the 1X sample buffer to help the detection of G proteins in co-IPs. Proteins were separated and transferred electrophoretically to a nitrocellulose membrane in cold 1X CAPS transfer buffer (10mM CAPS, pH 11, 10% methanol). Following the transfer, membranes were Ponceau stained and cut between 50kDa. Membranes were blocked for 1 hr in TBS with 0.1% Tween-20 (TBST) blocking solution with 5% milk on a shaker and washed 5 times for 5 mins with TBST on a shaker. Membranes were incubated overnight at 4°C with appropriate primary antibodies in TBS with 5% Milk and 0.2% Tween-20 on a shaker. Membranes were subsequently washed 5 times with TBST and incubated for 1 hr at room temperature with

appropriate secondary antibodies in TBS with 5% Milk and 0.2% Tween-20 on a shaker. Membranes were washed with 0.1% TBST for three times, 10 mins per wash, followed by two 15 mins washes with TBS. Using Western Lightning™ Chemiluminescence Reagent Plus (Perkin-Elmer) and Bio-rad Western blot imager, Western blots were developed.

Limit of detection. To estimate the number of IPs that would be necessary for MRM experiments, a limit of detection and quantitation (LOD/LOQ) experiment was performed using a dilution series of purified $G\beta_1\gamma_1$ from 1pg to 10ng. As described previously[7], targeted MRM methods were applied to the dilution series to estimate the LOD/LOQ for our studies. Quantitative Western blots were performed using a co-IP sample and analyzed for densitometry using Image J[8]

Heavy labeled peptide cocktail. A heavy labeled peptide cocktail was made as described previously[7]. In brief, using the mass spectrometry signal strength of co-IP samples, the peptide quantity was further adjusted to create a "heavy labeled peptide cocktail."

Quantitative MRM of Gβ and Gγ subunits. Co-IP samples containing Gβ and Gγ subunits were separated, digested, and analyzed by a TSQ Vantage triple quadrupole mass spectrometer (Thermo Scientific)[7]. Instead of purified $G\beta_5\gamma_2$ as a marker, PageRuler™ unstained low range protein ladder (ThermoFisher) was used to excise Gβ and Gγ bands.

Fig. S1. Estimation of the limit of detection for MRM experiments

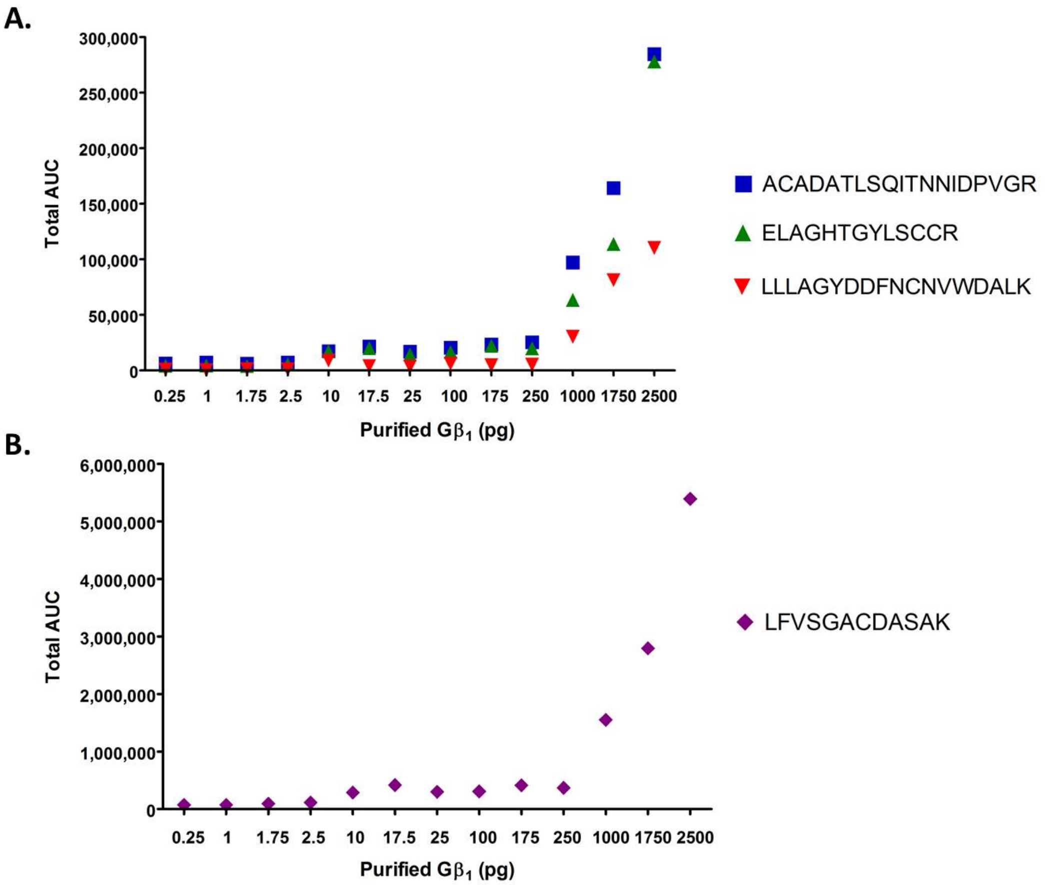

Total area under the curve (AUC) for $G\beta_1$ proteolytic peptides, ACADATLSQITNNIDPVGR, ELAGHTGYLSCCR, and LLAGYDDFNCNVWDALK (A) and LFVSGACDASAK (B), were monitored by MRM across a dilution series of purified $G\beta_1$.

Fig. S2. Co-immunoprecipitation of HA-α_{2a} adrenergic receptors and Gβγ.

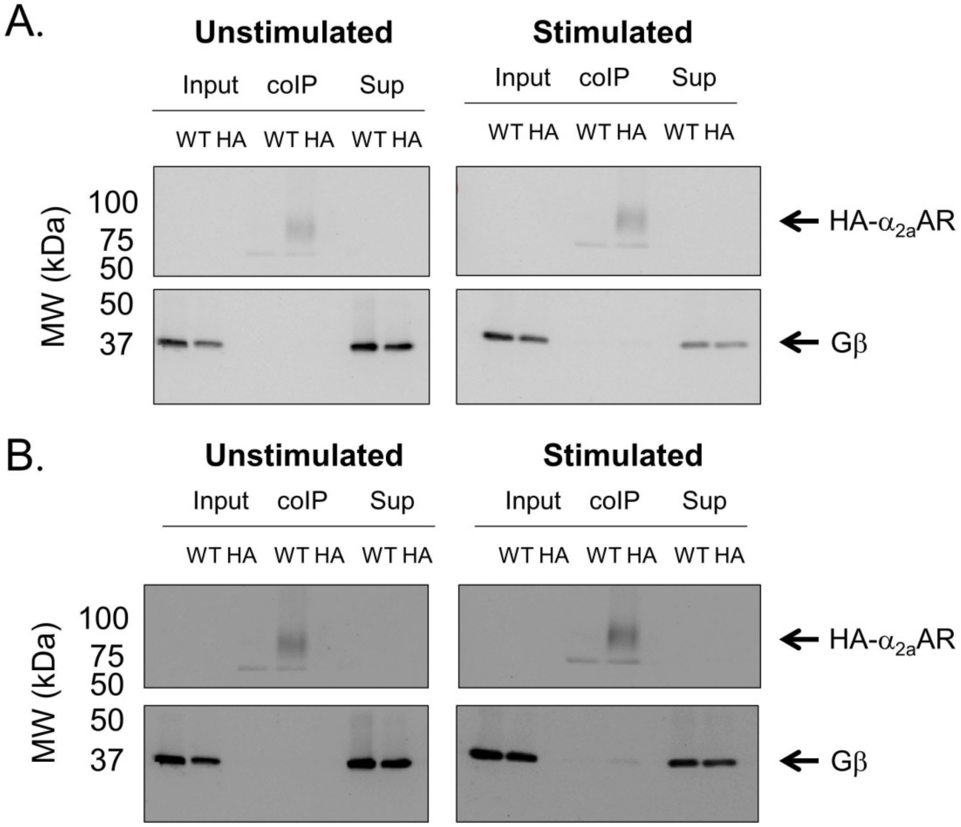

Multiple exposures of representative Western blot of coIP of the HA-α_{2a}AR and Gβ following the resuspension of synaptosomes with unstimulated or stimulated buffers (stimulated, 100μM epinephrine). Gels are cut out at 50kDa to separate receptor and Gβ blots. The exposure times are 100 (**A**) and 200secs (**B**), respectively. HA-α_{2a}AR is at ~75kDa while Gβ is ~33kDa. Sup: depleted supernatant

Fig. S3. Co-immunoprecipitation of FLAG-α_{2a} adrenergic receptors and Gβγ.

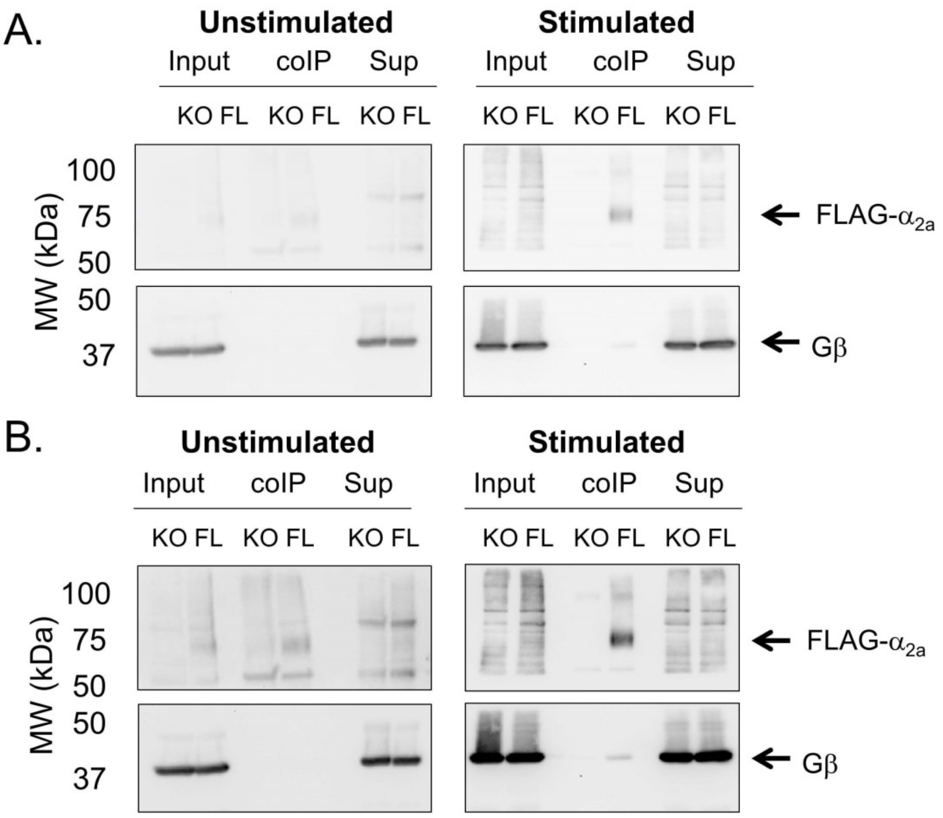

Multiple exposures of representative Western blot of coIP of the FLAG-α_{2a}AR and Gβ following the resuspension of synaptosomes with unstimulated or stimulated buffers (stimulated, 100μM epinephrine). Gels are cut out at 50kDa to separate receptor and Gβ blots. The exposure times of receptor blots are 30 (**A**) and 90 (**B**) secs, respectively. The exposure times of Gβ blots are 20 (**A**) and 60 (**B**) secs for FLAG-α_{2a}ARs coIP. FLAG-α_{2a}AR is at ~75kDa while Gβ is ~33kDa. Sup: depleted supernatant

Table S1. Proteolytic Gβ_1 peptides.

Name	Sequence position	Peptide Sequence	Precursor m/z	charge	Product ion m/z
**Gβ_1**	24-42	(K)ACADATLSQITNNIDPVGR(I)	1008.4944	2	428.26,543.29, 884.46, 985.51
	138-150	(R)ELAGHTGYLSCCR(F)	762.3401	2	641.28, 858.36, 915.38, 1016.43
	**198-209**	(R)LFVSGACDASAK(L)	613.2977	2	483.22,779.34, 866.37,965.44
	**284-301**	(R)LLLAGYDDFNCNVWDALK (A)	1064.0144	2	632.34, 894.39, 950.93, 1119.52

The LOD of our studies was estimated by using targeted MRM methods on serially diluted Gβ_1 peptides. Bolded peptides were the ones that were heavy labelled for the quantitative MRM analysis.

References

1 Lu, R. J. *et al.* Epitope-tagged Receptor Knock-in Mice Reveal That Differential Desensitization of alpha(2)-Adrenergic Responses Is because of Ligand-selective Internalization. *Journal of Biological Chemistry* **284**, 13233-13243, doi:DOI 10.1074/jbc.M807535200 (2009).

2 Gilsbach, R. & Hein, L. Are the pharmacology and physiology of alpha(2) adrenoceptors determined by alpha(2)-heteroreceptors and autoreceptors respectively? *British journal of pharmacology* **165**, 90-102, doi:10.1111/j.1476-5381.2011.01533.x (2012).

3 Gray, E. G. & Whittaker, V. P. The isolation of nerve endings from brain: an electron-microscopic study of cell fragments derived by homogenization and centrifugation. *Journal of anatomy* **96**, 79-88 (1962).

4 Whittaker, V. P., Michaelson, I. A. & Kirkland, R. J. The separation of synaptic vesicles from nerve-ending particles ('synaptosomes'). *The Biochemical journal* **90**, 293-303 (1964).

5 Betke, K. M. *et al.* Differential localization of G protein betagamma subunits. *Biochemistry* **53**, 2329-2343, doi:10.1021/bi500091p (2014).

6 Michael J Hudspith, P. J. S., and Rajesh Munglani. in *Foundations of Anesthesia* Vol. 2nd Edition (ed BS Hugh C. Hemmings, MD, PhD and Phillip M. Hopkins, MB, BS, MD, FRCA) Ch. 23, (Elsevier Mosby, 2006).

7 Yim, Y. Y. *et al.* Quantitative Multiple-Reaction Monitoring Proteomic Analysis of Gbeta and Ggamma Subunits in C57Bl6/J Brain Synaptosomes. *Biochemistry* **56**, 5405-5416, doi:10.1021/acs.biochem.7b00433 (2017).

8 Schneider, C. A., Rasband, W. S. & Eliceiri, K. W. NIH Image to ImageJ: 25 years of image analysis. *Nat Methods* **9**, 671-675 (2012).
